# Reply to Theodorou et al. Comment on “Umemoto et al. Management of Migraine-Associated Vestibulocochlear Disorders. *Audiol. Res.* 2023, *13*, 528–545”

**DOI:** 10.3390/audiolres14010016

**Published:** 2024-02-07

**Authors:** Najva Mazhari, Karen Tawk, Kayla K. Umemoto, Mehdi Abouzari, Hamid R. Djalilian

**Affiliations:** Department of Otolaryngology-Head and Neck Surgery, University of California, Irvine, CA 92697, USA

We thank the authors for their insightful and thoughtful commentary on our recent publication [1]. The authors advocate the use of a specific magnetic resonance imaging (MRI) protocol that employs 3D-constructive interference in a steady state or a similar fast gradient echo pulse sequence as a complement to routine brain MRI for investigating the inner ear in patients with migraine. Moreover, the authors emphasize that this readily accessible sequence provides high-resolution images and multiplanar reconstructions, allowing for the visualization of fine anatomical structures and nerves without the drawbacks associated with the use of gadolinium. 

In line with the points raised in the correspondence with our paper, we conducted a study using T2-weighted MRI to investigate whether migraine-related aural fullness correlates to mastoid T2 hyperintensity in MRI. Our findings have unveiled a notably higher incidence of T2 hyperintensities among patients with migraine headaches in comparison to those with headaches not meeting the criteria outlined in ICHD-3 (unpublished data). In addition, we have authored a manuscript that compares white matter hyperintensities in T2-weighted MRI in patients with sudden sensorineural hearing loss (SSNHL). Our investigation has shown a significantly higher likelihood of periventricular and deep white matter hyperintensities in patients with SSNHL when compared to age-matched controls (unpublished data). A meta-analysis conducted by Swartz et al. has provided evidence that individuals afflicted by migraine face an elevated risk of exhibiting white matter abnormalities in MRI as opposed to those who do not experience migraine [2]. 

In the spectrum of migraine-related disorders, Meniere’s disease (MD) has been the subject of extensive MRI studies, revealing distinctive radiological features such as a shorter MRI-PP distance (distance between the vertical part of the posterior semicircular canal and the posterior fossa) and poorer MRI-VA visibility (visibility of vestibular aqueduct) in unilateral MD. These distinct findings suggest potential inner ear anatomical variations linked to endolymphatic duct and vestibular aqueduct hypoplasia. In fact, various MRI techniques, including intratympanic gadolinium contrast-enhanced 3D-real-IR MRI, have been performed to attempt to distinguish MD and vestibular migraine (VM) based on characteristic pathological changes, despite both conditions sharing similarities in vestibular dysfunction and transduction pathways [3]. Furthermore, functional MRI has shown promise for diagnosing migraine. Li et al. found that patients with VM present modifications in functional connectivity within sensorimotor networks, particularly in the medial cingulate gyrus and paracingulate gyrus. These alterations may contribute to patients’ heightened sensitivity to sensory stimuli such as photophobia and phonophobia. Additionally, they have noted changes in connectivity between the auditory network and other brain networks, indicating that VM may be characterized by functional brain alterations rather than structural abnormalities [4]. 

As you point out, the integration of MRI into our diagnostic process holds the potential for significant advancements in our understanding of migraine and its related symptoms and disorders. However, it is important to acknowledge that, at this time, conclusive insights into how MRI findings could influence our treatment approach in migraine patients (where clinical diagnosis continues to play a pivotal role) remain elusive. Our routine protocol for MRI in patients with vertigo is a non-contrast MRI of the internal auditory canals (IAC), which includes CISS sequence in axial, coronal, and sagittal directions. In addition, our IAC MRI protocol includes an axial brain T2 sequence that can show other pathologies of the brain and the T2 white matter changes that are evidence of previous migraine. Though MRI can rule out other pathologies, its diagnostic yield is generally low. Further research is required to establish the full potential of MRI and other imaging modalities in more effective treatment strategies.
